# Modulation of immune cell infiltrate with sunitinib to improve anti-PD1 therapy in preclinical tumor model

**DOI:** 10.1186/2051-1426-3-S2-P310

**Published:** 2015-11-04

**Authors:** Patricia Rayman, C Marcela Diaz-Montero, Yu Yang, Brian Rini, Paul Elson, James Finke

**Affiliations:** 1 Cleveland Clinic Foundation, Cleveland, OH, USA; 2 Cleveland Clinic, Cleveland, OH, USA

## 

Combined therapy using the checkpoint blockade anti-PD1 antibody and the TKI, sunitinib, has been investigated in metastatic renal cell carcinoma (mRCC). Here we examined the impact of sunitinib plus anti-PD1 therapy on tumor growth in the renal cell carcinoma mouse model RENCA. Treatment was initiated on day 5 after implantation of tumor (0.5 million) subcutaneously and consisted of oral gavage with sunitinib (40mg/kg) given daily for 21 days while anti-PD-1 antibody (BioXcell) was given i.p (200ug/100ul) twice a week for three 3 weeks. Treatment groups included sunitinib, anti-PD-1 and both agents while untreated RENCA bearing mice served as controls. Because sunitinib is so effective at reducing tumor growth in the RENCA model, analysis of immune changes in the tumor were assessed in mice with larger and more established tumors (treatment was initiated on day 17, given at the same doses and methods for 7 days). Sunitinib consistently induced a 54% reduction in tumor volume if treatment started on day 17, and >90% if started Day 5, which was not sustained once drug therapy ended. Anti-PD-1 antibody monotherapy caused variable reduction in tumor volume and modestly prolonged survival but only the combination therapy was effective at inducing complete tumor regression in the mice. FACS analysis of single cell suspension after tumor digest revealed that sunitinib alone significantly reduced granulocytic (CD11b+Ly6G+Ly6Clow) and monocytic (CD11b+Ly6G-Ly6Chi) myeloid derived suppressor cells (MDSC) by 52% and 68%, respectively. With combination treatment MDSC were reduced by 74% (G-MDSC) and 94% (M-MDSC) respectively from untreated tumors. In contrast anti-PD-1 antibody monotherapy had no effect on MDSCs levels. FACS studies further revealed sunitinib monotherapy caused an immune cell infiltrate that was composed predominately of CD8+ T cells (38%) compared to PD-1 antibody treatment (6%). A high percentage of the CD8+ T cells from the sunitinib and sunitinib/anti-PD1-treated mice expressed CD107a (a lysomal-associated membrane protein known to be involved in lytic destruction of tumor target cells) (44% and 60%), compared to untreated and anti-PD1 treated mice (3% and 7% respectively). Combination with sunitinib and PD-1 antibody treatment not only increases the infiltrate of CD8+ T cells but also their frequency of actively cytolytic CD8+ T cells as evidenced by their increased frequency of CD107a expressing cells, suggesting a reverse of immunosuppression by MDSCs. Supported by Pfizer and NIH RO1 CA168488.

